# The career pathways of new family physicians in South Africa from 2008 to 2022

**DOI:** 10.4102/safp.v66i1.5904

**Published:** 2024-05-08

**Authors:** Gabby Jacobs, Robert J. Mash

**Affiliations:** 1 Division of Family Medicine and Primary Care, Faculty of Medicine and Health Sciences, Stellenbosch University, Cape Town, South Africa

**Keywords:** family physicians, career pathway, career choices, human resources, human resource development

## Abstract

**Background:**

Family medicine has trained specialist family physicians in South Africa since 2008, but not investigated their career pathways. The study aimed to determine the career pathways of newly qualified family physicians between 2008 and 2022.

**Methods:**

A cross-sectional descriptive survey of all 186 family physicians via an electronic questionnaire.

**Results:**

Response rate was 44.6% (83/186). Overall, 9.6% emigrated, 10.8% were no longer practising, and 79.5% were still practising in South Africa. Of the latter, 14.5% were in the private sector, 55.4% in the public sector and 9.6% in both. Of those in the public sector, 33.7% were in specialist family physician posts, 12% in medical officer posts, 4.8% in managerial positions and 4.8% in academic positions. Issues relating to safety and security were important to those working in both sectors and relationships with colleagues in the clinical team, to those in the public sector. Overall, participants practised near or within their province of training and were not equitably distributed.

**Conclusion:**

Only a third of graduates were in specialist family physician posts in the public sector. Attention needs to be given to retaining more graduates in such posts to achieve the goals of the national position paper. The proportion in the private sector was lower than expected. The reasons for no longer practising medicine should be further explored.

**Contribution:**

This is the first study on the career pathways of family physicians in South Africa since the new speciality was created. Understanding these pathways will assist with human resources for health planning.

## Introduction

South Africa’s National Development Plan of 2030 affirms that the foundation of the health service is the district health system, with a specific focus on high-quality primary health care.^1^ In aiming to achieve this, many initiatives have attempted to improve the district health system, such as the introduction of ward-based outreach teams and development of district clinical specialist teams.^2^ The introduction of family physicians as a speciality can be viewed as another such intervention.^3^

Family physicians are specialists in family medicine and have the potential to strengthen the district health services through their roles of clinician and consultant, capacity builder and clinical trainer, and leader of clinical governance.^4^ Family physicians may fulfil these roles as part of community-oriented primary care, in district hospitals or district clinical specialist teams. Family physicians are effective in these roles and having a family physician is associated with improved health indicators.^3^

Family medicine was recognised as a speciality in South Africa in 2007, which led to the establishment of formal postgraduate registrar training programmes. The 4-year training programme consists of workplace-based training and assessment in primary health care, district hospitals and sometimes other regional facilities, as part of a Master of Medicine degree through nine South African universities. The degree also includes an academic programme, research assignment and culminates in a national fellowship examination through the Colleges of Medicine of South Africa (CMSA).

As of 2019, the Health Professions Council of South Africa (HPCSA) register documented 194 new specialist family physicians since 2007 and 775 grandfathered family physicians, those who qualified as family physicians from older programmes.^5^ The CMSA graduate list, which is published in their Transactions Journal, identifies 186 graduates from the family medicine registrar programme as of 2022. Bureaucratic processes, different time periods and changing registration criteria as the discipline was established may explain the discrepancy between HPCSA and CMSA numbers. Currently, the average number of new registrars per year across all nine programmes is 48, the average number of total registrars is 177, and the average number of annual graduates is 27 (personal communication, South African Academy of Family Physicians [SAAFP] data 2008–2022).

The HPCSA database does not provide accurate information on the distribution of family physicians, or what exactly they are doing after registration. Human resources for health planning requires this more textured information. The HPCSA address data suggest that family physicians are concentrated in Gauteng, Western Cape and KwaZulu-Natal, despite these provinces only housing five of the nine training programmes.^5^ However, the registered addresses and actual locations may differ. Internationally, retention of the workforce in rural areas has been linked to training in rural areas.^6^ In contrast, the choice of urban practice is often driven by increased professional development opportunities and family factors, including spousal employment opportunities.^7,8,9^ It is thought that the majority of family physicians remain near their training site, which suggests that decentralised training may be part of the solution to uneven distribution.^7,8,9^

In the public sector, the national position paper on family physicians has a goal of one family physician for every district hospital, community health centre and subdistrict without a health centre.^4^ To achieve this goal, over 10 years, the number of family medicine registrar posts must be doubled, the throughput rate increased to 70%, and 60% of graduates retained in public sector family physician posts.^4^ Retention of family physicians in the public sector and the proportion emigrating are currently unknown. It is important to survey graduates to obtain a more accurate picture of their career pathways. This can assist the discipline with achieving their goals and the National Department of Health with human resources for health planning.^10^

The aim was to determine the career pathways of family physicians, after graduating from South African specialist training programmes. Specific objectives were to identify their geographical distribution, public versus private sector distribution, types of posts occupied, relationship between training sites and locations of practice, and the factors influencing their career paths.

## Methods

### Study design

This study was a cross-sectional descriptive survey.

### Study setting

Training of family physicians is offered through nine programmes, each linked to a university. The nine universities (Sefako Makgatho University, University of Limpopo, Stellenbosch University, University of Cape Town, University of KwaZulu-Natal, University of Pretoria, University of the Free State, University of the Witwatersrand, and Walter Sisulu University) have training sites in all nine provinces, in both rural and urban settings. After graduating, specialist family physicians can work in both the private and public health care sectors or continue in academia or research. In the public sector, there is provision for specialist posts at primary and district levels of care within the district health system; however, specialist family physicians can occupy a medical officer post after graduation. Medical officers are general practitioners, without further specialist training who work within the public sector. In the private sector, family physicians are not compensated as specialists and their scope of practice is also not fully recognised by medical aid schemes.^4^

### Study population

Family physicians who completed the training through the CMSA between 2008 and 2022 were included. Prospective participants were identified from the CMSA graduate lists published online. According to this, the study population included 186 participants, and there were no exclusion criteria. The whole study population was invited to participate and there was no sampling.

### Data collection

A questionnaire was developed by the researcher in which potential variables influencing career pathways of family physicians were identified from current literature. The variables of geographical distribution, public versus private sector distribution, types of posts occupied, relationship between training sites and locations of practice, and the workplace and personal factors influencing their career paths were identified and questions created. The questionnaire included closed-ended questions for demographics, Likert scale ratings of personal and workplace factors, and a free text submission of their employment history post-graduation.

The questionnaire was validated by a panel of four experts in family medicine and primary care research in the South African context. The panel recommended changes to the number and clarity of items and approved the final version. The questionnaire was piloted among six family physicians in the Western Cape. The questionnaire was then designed in REDCap, and an invitation to participate was distributed via email with the aid of the SAAFP with follow-up reminder emails sent 4 weeks after the initial email. The heads of university departments also aided in distribution of reminder emails. The communication for this research was in English as CMSA exams are conducted in English and so proficiency in the language is innate in the study population.

### Data analysis

Data were exported from REDCap into the Statistical Package for Social Sciences version 28. Data were checked for errors during the initial descriptive analysis and corrected if necessary. Data analysis was conducted by the researcher and her supervisor together. Additional variables were created from the text fields in the questionnaire and categorised for later analysis: title of first job and occupancy of family physician post during the career pathway.

In 2015, the programme under the University of Limpopo was separated into two programmes: one remained under the University of Limpopo and the other was under Sefako Makgatho Health Sciences University (SMU). To avoid confusion, these two programmes were grouped together as one category.

Descriptive analysis reported categorical data as frequencies and percentages. Numerical data were reported as the median and interquartile range, or the mean and standard deviation, depending on their distribution.

For inferential analysis, the dependent variables included sector of practice, retention in South Africa, current occupancy of a family physician post and province of current practice. Independent variables were gender, classification of training as metro or rural, university of training, province of training, personal and workplace factors.

The relationship between independent and dependent variables was then analysed by means of cross-tabulation and the Chi-square test, with a *p*-value of < 0.05 being regarded as statistically significant. Analysis of age as an independent numerical variable in relation to dependent categorical variables was done using the analysis of variance (ANOVA) test.

### Ethical considerations

The study was approved by the Health Research Ethics Committee at Stellenbosch University (S21/10/206), and the National Education and Training Committee of the SAAFP gave permission for the Academy to assist with the study.

## Results

Overall, 83 of the potential 186 respondents completed the questionnaire giving a response rate of 44.6%. The mean age of participants was 43.8 years (s.d. = 6.1) with 59% being between the ages of 40 and 49 years.

Table 1 shows the characteristics of participants. Most participants were male (65.1%), spoke either Afrikaans or English (85.5%) and were recently qualified (2018–2022, 51.8%). Most respondents (78.4%) trained in the Western Cape, KwaZulu-Natal and Gauteng and were from Stellenbosch, Cape Town, Witwatersrand and KwaZulu-Natal universities (80.2%). The majority had trained in metropolitan sites (63.9%).

**TABLE 1:** Characteristics of participants.

Characteristics	*n*	%
**Gender (*N* = 83)**		
Male	54	65.1
Female	28	33.7
Other	1	1.2
**Age (years) (*N* = 83)**		
30–39	21	25.3
40–49	49	59.0
50–59	11	13.3
60–69	2	2.4
**Home language (*N* = 83)**		
English	49	59.0
Afrikaans	22	26.5
African language	5	6.0
Other	7	8.5
**Year of qualification (*N* = 82)**		
2008–2012	10	12.0
2013–2017	29	34.9
2018–2022	43	51.8
**University of post graduate training (*N* = 82)**		
Sefako Makgatho University/University of Limpopo	1	1.2
Stellenbosch University	26	31.3
University of Cape Town	11	13.3
University of KwaZulu-Natal	17	20.5
University of Pretoria	7	8.4
University of the Free State	3	3.6
University of the Witwatersrand	13	15.7
Walter Sisulu University	4	4.8
**Province of postgraduate training (*N* = 83)**		
Eastern Cape	4	4.8
Free State	2	2.4
Gauteng	11	13.3
KwaZulu-Natal	17	20.5
Mpumalanga	2	2.4
Limpopo	0	0.0
Northern Cape	1	1.2
North West	9	10.8
Western Cape	37	44.6
**Training site classification (*N* = 83)**		
Rural	30	36.1
Metropolitan	53	63.9

Table 2 shows the distribution and current practice of participants. Overall, 20.5% were no longer practising in South Africa, although this included family physicians who were in South Africa but not in practice (10.8%) and those who had emigrated abroad (9.6%). Most participants remained in the district health system (74.6%) and in the public sector (63.9%). Job title distribution showed that 53% reported being in specialist family physician positions, while 8.4% held managerial titles and 6% occupied posts as lecturers or academics, with 32.6% in medical officer or general practitioner posts. The Western Cape had the largest proportion of family physicians (44.6%), followed by KwaZulu-Natal (18.1%), Gauteng and North West (8.4% each) and the remaining five provinces only housing 10.8% of family physicians collectively.

**TABLE 2:** Distribution and current practice of participants.

Variables	*n*	%
**Current practice location (*N* = 82)**		
Not practising in South Africa	17	20.5
Eastern Cape	4	4.8
Free State	1	1.2
Gauteng	7	8.4
KwaZulu-Natal	15	18.1
Limpopo	1	1.2
Mpumalanga	2	2.4
Northern Cape	1	1.2
North West	7	8.4
Western Cape	37	44.6
**Current job title (*N* = 83)**		
Medical officer	13	15.7
General practitioner	14	16.9
Specialist family physician	44	53.0
Clinical manager	5	6.0
Facility manager	1	1.2
Chief operations officer	1	1.2
Lecturer or academic	5	6.0
**Current facility type (*N* = 83)**		
Primary care/general practice	39	47.0
District hospital	20	24.0
Regional hospital	6	7.2
Tertiary hospital	3	3.6
District clinical specialist team	1	1.2
Subdistrict specialist	2	2.4
University	5	6.0
Specialised hospital	1	1.2
Unemployed	2	2.4
Unknown	2	2.4
NGO	1	1.2
Other	1	1.2
**Sector of practice (*N* = 83)**		
Public	53	63.9
Private	18	21.7
Both	12	14.5
**First job title after graduating (*N* = 83)**		
Family physician	54	65.1
Nonfamily physician	29	34.9

**FIGURE 1:**
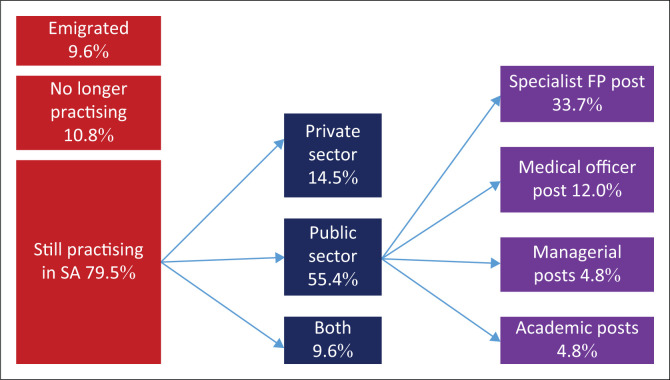
Distribution of family physicians practising in South Africa (*N* = 83).

As shown in Figure 1, out of the 66 (79.5%) family physicians who are still practising in South Africa, 46 (55.4%) remain in the public sector, 12 (14.5%) in the private sector and 8 (9.6%) in both. In the public sector, 33.7% of all family physicians were in specialist posts, 12.0% in medical officer posts, 4.8% in managerial posts and 4.8% in academic posts.

Table 3 reflects personal and workplace factors that influenced their career decisions. From this, it is noted that four personal factors (needs of spouse, needs of children, issues related to safety and security, and salary or remuneration) were ranked as the most important in determining career choices. The relationship with the colleagues in the clinical team was the strongest workplace factor to influence career choice, closely followed by the opportunity for promotion.

**TABLE 3:** Personal and workplace factors influencing career pathways of family physicians.

Factors	*n*	%
**Personal factors (*N* = 83)**		
Needs of spouse	37	44.0
Needs of children	36	43.4
Issues related to safety and security	34	41.0
Salary or remuneration	34	41.0
Issues related to community	21	25.3
Issues related to travel	15	18.1
Needs of extended family	11	13.3
Needs of social network	6	7.2
**Workplace factors (*N* = 83)**		
Relationship with colleagues in the clinical team	53	63.9
Opportunities for promotion	49	59.0
Recognition and acknowledgement	45	54.2
Amount of clinical support	44	53.0
Job content	41	53.0
Relationships with management	38	45.8
Overtime requirements	38	45.8
Workload	36	43.4
Flexible working hours	32	38.6

Table 4 shows the association of factors with a career in the public or private sector. Participants who noted issues regarding safety and security to be an influencing factor were more likely to work in both sectors (*p* = 0.02). Participants in the public sector were more likely to consider the relationship with colleagues in the clinical team to be an important workplace factor (*p* < 0.005) when compared to the private sector or both. There were no other significant associations. None of the factors listed in Table 4 were significantly associated with the decision to emigrate or with occupying a family physician post in the public sector.

**TABLE 4:** Factors associated with public versus private sector career pathways.

Factors	Public sector (*N* = 53)		Private sector (*N* = 18)		Both (*N* = 12)		*p*-value
	*n*	%	*n*	%	*n*	%	
**Gender**	-	-	-	-	-	-	0.993
Male	35	66.0	11	61.1	8	66.7	-
Female	18	34.0	6	33.3	4	33.3	-
**Training site**							0.823
Metro training site	33	62.3	12	66.7	8	66.7	-
Rural training site	20	37.7	6	33.3	4	33.3	-
**University of training**	-	-	-	-	-	-	0.209
Sefako Makgatho University/University of Limpopo	0	0.0	0	0.0	1	8.3	-
Stellenbosch University	20	37.7	3	16.7	3	25.0	-
University of Cape Town	8	15.1	2	11.1	1	8.3	-
University of KwaZulu-Natal	7	13.2	7	38.9	3	25.0	-
University of Pretoria	3	5.7	3	16.7	1	8.3	-
University of the Free State	3	5.7	0	0.0	0	0.0	-
University of the Witwatersrand	8	15.1	2	11.1	3	25.0	-
Walter Sisulu University	3	5.7	1	5.6	0	0.0	-
**Province of training**	-	-	-	-	-	-	0.126
Eastern Cape	3	5.7	1	5.6	0	0.0	-
Free State	2	3.8	0	0.0	0	0.0	-
Gauteng	8	15.1	1	5.6	2	16.7	-
KwaZulu-Natal	7	13.2	7	38.9	3	25.0	-
Mpumalanga	0	0.0	2	11.1	0	0.0	-
Limpopo	0	0.0	0	0.0	0	0.0	-
Northern Cape	1	1.9	0	0.0	0	0.0	-
North West	4	7.6	2	11.1	3	25.0	-
Western Cape	28	52.8	5	27.8	4	33.3	-
**Workplace factors**	-	-	-	-	-	-	-
Relationship with colleagues in the clinical team	41	77.6	8	44.4	4	33.3	0.005
Amount of clinical support	29	54.7	7	38.9	8	66.7	0.563
Working environment	21	39.6	10	55.6	4	33.3	0.765
Job content	31	58.5	12	66.7	1	8.3	0.130
Relationships with management	27	50.9	6	33.3	5	41.7	0.453
Recognition and acknowledgement	30	56.6	10	55.6	5	41.7	0.283
Opportunities for promotion	35	66.0	9	50.0	5	41.7	0.415
Overtime requirements	27	50.9	6	33.3	5	41.7	0.465
Workload	24	45.3	5	27.8	7	58.3	0.187
Flexible working hours	15	28.3	9	50.0	8	66.7	0.091
**Personal factors**	-	-	-	-	-	-	-
Needs of spouse	28	52.8	6	33.3	3	25.0	0.120
Needs of children	23	43.4	8	44.4	5	41.7	0.989
Issues related to safety and security	17	32.0	8	44.4	9	75.0	0.023
Salary or remuneration	20	37.7	6	33.3	8	66.7	0.1391
Issues related to community	15	28.3	2	11.1	4	33.3	0.275
Issues related to travel	11	20.8	2	11.1	2	16.7	0.650
Needs of extended family	7	13.2	3	16.7	1	8.3	0.803
Needs of/for social network	5	9.4	0	0.0	1	8.3	0.405
**First job title after graduation**	-	-	-	-	-	-	0.208
Family physician	32	60.4	12	66.7	10	83.3	-
Nonfamily physician	16	30.2	3	16.7	10	83.3	-

Table 5 compares the university where participants trained to the province where they currently practise. The blank cells had no data and were left blank to make the table easier to read. Most family physicians remained in the provinces where their university had a training programme, but there was a substantial number that relocated to the Western Cape. Substantial numbers of graduates from the University of the Witwatersrand and KwaZulu-Natal had also migrated.

**TABLE 5:** Percentage of participants currently practising in a specific province by university of training.

University of training	EC		FS		Gauteng		KZN		LP		MP		NC		NW		WC		Out of RSA		Total		*p*-value
	*n*	%	*n*	%	*n*	%	*n*	%	*n*	%	*n*	%	*n*	%	*n*	%	*n*	%	*n*	%	*n*	%	
SU	-	-	-	-	-	-	-	-	1	3.8	-	-	-	-	-	-	25	96.2	-	-	26	100.0	-
UCT	1	9.1	-	-	-	-	-	-	-	-	-	-	-	-	-	-	9	81.8	1	9.1	11	100.0	-
UFS	-	-	1	33.3	-	-	-	-	-	-	-	-	1	33.3	-	-	1	33.3	-	-	3	100.0	-
UKZN	-	-	-	-	-	-	14	82.4	-	-	-	-	-	-	-	-	-	-	3	17.6	17	100.0	-
UP	-	-	-	-	4	57.1	-	-	-	-	2	28.6	-	-	-	-	1	14.3	-	-	7	100.0	-
Wits	-	-	-	-	1	7.7	-	-	-	-	-	-	-	-	7	53.8	1	7.7	4	30.8	13	100.0	-
WSU	3	75.0	-	-	-	-	1	25.0	-	-	-	-	-	-	-	-	-	-	-	-	4	100.0	-
SMU/UL	-	-	-	-	1	100.0	-	-	-	-	-	-	-	-	-	-	-	-	-	-	1	100.0	-
**Total**	**4**	**4.9**	**1**	**1.2**	**6**	**7.3**	**15**	**18.3**	**1.2**	**1.2**	**2**	**2.4**	**1**	**1.2**	**7**	**8.5**	**37**	**45.1**	**8**	**9.8**	**82**	**100.0**	**< 0.001**

SU, Stellenbosch university; UCT, University of Cape Town; UFS, University of Free State; UKZN, University of KwaZulu-Natal; UP, University of Pretoria; Wits, University of the Witwatersrand; WSU, Walter Sisulu University; SMU/UL, Sefako Makgatho University/University of Limpopo.

EC, Eastern Cape; FS, Free State; KZN, KwaZulu-Natal; LP, Limpopo; MP, Mpumalanga; NC, Northern Cape; NW, North West; WC, Western Cape; RSA, Republic of South Africa.

## Discussion

### Summary of key findings

Overall, 79.5% of participants remain practising within South Africa, while 10.8% were not working as medical practitioners and 9.6% emigrated to other countries. Out of those practising in South Africa, 55.4% worked within the public sector, 14.5% in the private sector and 9.6% in both. Out of those in the public sector, only 33.7% were in specialist family physician posts, while 12.0% were in medical officer posts and the rest in managerial or academic positions. Family physicians that valued relationships within a clinical team were more likely to remain in the public sector, while those concerned about safety and security were more likely to move towards the private sector. Graduates were mostly retained in the provinces where they trained, although the Western Cape attracted substantial numbers.

### Discussion of key findings

It is worrying that 12% of specialists were occupying medical officer posts. This could be because many provinces had failed to create enough family physician posts or because some family physicians preferred to remain in a specific location, rather than relocate to where posts were available. Family physicians who work in medical officer posts may not be able to make full use of their training and may not make their expected contribution to strengthening health systems.^11^ This is because the job description of medical officers and their roles differ from those of family physicians.^12^

Family physicians are also being appointed as managers within health facilities, and in some areas these posts are more available than clinical family physician posts. Family physicians are trained as clinicians and not managers but are increasingly being appointed as clinical managers (personal communication, chief directors, Western Cape), and this may be a future strategy to deploy them in the provinces (personal communication, National Department of Health).

A study on the retention of medical officers in the district health system in the Western Cape indicated that personal factors pertaining to social support, needs of children and needs of spouse were important to retaining them in public service.^13^ While participants saw these same factors as important in influencing their career pathway, they were not statistically associated with the type of health sector they worked in. Safety and security appeared to be an important factor in family physicians more inclined towards working in the private sector and this was also noted as an important consideration for community service doctors in their career decision-making (Low & Mash).^14^ Medical officers, already working in the public sector, did not regard this as a significant factor in retention.^15^

Being part of a cohesive team with strong supportive relationships was also seen as important in the retention of medical officers.^13,15^ The public sector may be more predisposed towards working in multidisciplinary teams with a mix of specialist, nonspecialist and nonprofessional colleagues. The private sector regulations and remuneration model make it difficult for practices to register in this way and reduce opportunities for such teamwork in general practice.^4^ By creating supportive clinical environments and fostering good working relationships, we could encourage recruitment and retention within the public sector.

Only 14.5% completely entered the private sector, which is less than the 29% of all family physicians on the HPCSA register thought to be in the private sector.^5^ The private sector does not fully recognise the scope of practice and specialist status of family physicians, particularly in terms of the tariffs, and this acts as a disincentive to work in the private sector. It is also difficult for family physicians to work in collaborative practices due to the regulations on mixing specialists and nonspecialists in one practice.^4^

In keeping with international literature, participants mostly remained within the provincial footprint of their training university.^7,8,9^ Deployment of family physicians throughout the country may therefore require training complexes and registrar posts in all the provinces, as well as the availability of posts. Similarly, we know that training in rural areas is a factor in recruiting and retaining family physicians in rural and remote settings.^7,8,9^ All provinces have at least one training complex but vary considerably in the availability of registrar posts. Some provinces need to increase the number of training complexes (e.g. Northern Cape), while others need to commit to funding registrar posts (e.g. KwaZulu-Natal). To achieve the goals of the national position paper, there is a need to double the number of registrar posts, improve the training throughout and retain 60% in the public sector in family physician posts (currently only 34%).

The geographical distribution of family physicians across South Africa, however, was found to be uneven, both in this study and other literature.^5^ The greatest movement between provinces was to the Western Cape, as this province has a track record of creating posts throughout the district health services.^16^ Most family physicians on the HPCSA register reside in Western Cape, Gauteng and KwaZulu-Natal. The distribution varies from 0.05 per 10 000 population in Limpopo to 0.3 per 10 000 in Western Cape.^5^ The findings here suggest that recent graduates are also skewed towards the Western Cape and KwaZulu-Natal.

Participants from Witwatersrand and University of KwaZulu-Natal showed the highest proportion of graduates working outside of South Africa. This could be due to training of foreign doctors with the intention of emigration after training, or a lack of posts. When the speciality was being established, not all programmes restricted registrar posts to South African citizens or permanent residents, and initially a few foreign doctors were accepted, although this is no longer the case.

The loss of family physicians from the medical profession within South Africa was more than the loss to emigration, and this is a worrying trend. High rates of burnout among public sector doctors could account for some of these losses,^17,18^ and some may be temporary due to parenting or family responsibilities.

### Limitations

The main limitation of this study was a low response rate with a risk of selection bias. Some graduates may not have been reached if they were no longer members of the SAAFP or their contact details were outdated. Given the increased difficulty of contacting graduates who left the country and their anticipated lower motivation to complete the questionnaire, the study likely underestimates the number of graduates who have left South Africa. Similarly, it may have been easier to reach family physicians within the public sector versus the private sector. Comparing the profile of respondents to the SAAFP records, it appears that graduates from SMU and Free State are underrepresented, while those from the University of Cape Town, Wits and KwaZulu-Natal are slightly overrepresented.

### Recommendations

The following recommendations can be made:

- More family physician posts are needed in the public sector to avoid family physicians working in medical officer posts and to achieve the 60% retention rate recommended by the national position paper. Similarly, each province needs sufficient training capacity to supply family physicians, including rural training programmes. There needs to be attention to both the supply and equitable distribution of family physicians to strengthen district health services.
- Employment as managers within the public sector needs to be monitored and further studied. On the one hand, this may create more cohesive and well-trained leadership and provide opportunities for graduates; on the other hand, it is not the intention of family medicine to train managers.
- Attention should be given to creating cohesive functional clinical teams that attract and retain family physicians in the public sector, while also ensuring that safety and security issues are addressed.
- Further research should explore why 10.8% of graduates are in South Africa, but no longer practising family medicine.
- Qualitative research may also help to explore the decision-making processes in the career choices of newly qualified family physicians.

## Conclusion

Most family physicians in this study work within the public sector after graduating. However, there remains 12% who do so, but not as the specialist they have been trained to be. More specialist posts need to be created to promote equitable distribution of family physicians across the country and to achieve the human resource goals of the SAAFP’s position paper. Training programmes can help in future equitable distribution by expanding intake of registrars at universities supplying provinces with lower numbers of family physicians. This, along with improved safety and security as well as good relationships within the clinical team, can influence the career pathways and strengthen the district health system. This study informs human resources for health policy and sets a basis for more quantitative and qualitative work in the field to better understand career pathways of family physicians.
